# Nerve Killing

**Published:** 1847-10

**Authors:** M. Rogers


### Nerve Killing.

*Extract from the Introductory Lecture in the Ohio College of Dental Surgery, at the opening of the Session of* 1846–7, *By* M. Rogers, M. D., *Professor of Dental Pathology and Therapeutics.*

When a new system of practice is introduced, the members of the profession should be able to examine it, and test its claims by the unerring principles of science. If it transgress a fixed law of the animal economy, it must fail, however plausible.

A few years ago the practice of destroying the nerve of an aching tooth with arsenic, cobalt or nitrate of silver was promulgated, and a practice set up by a certain class of practitioners of filling the tooth, with assurance to the patient that it would be free from pain afterwards, and as useful as ever. There are so many evils clustering around this fallacious doctrine that I propose to devote the remainder of this discourse to its examination.

To be clearly understood, a brief description of the structure of the teeth will be necessary.

That portion of the tooth above the gum is void of sensibility from the covering of a dense chrystalized substance, called the enamel. The rest of the solid part of the tooth is ivory or bone, dense and susceptible of a fine polish; yet when viewed with a microscope, equal to 2 or 300 diameters, innumerable little tubes are seen passing through every part of it. In the centre of the tooth is a cavity or canal, passing from the point of the fang to near the end above the gum. From this canal, as a centre, these little tubes all radiate to every part of the tooth.

A nerve, artery and vein enter this canal at the end of the fang, and expand into a kind of pulp, till it is entirely filled; from this pulp nerves and vessels enter these minute tubes, and extend to every portion of the ivory, till they terminate on the inner surface of the enamel. This is very manifest whenever the enamel is removed or the ivory perforated, even by a very finely pointed instrument, by its producing a very pungent thrill of pain.

The fifth pair of nerves arising from the base of the brain is immediately distributed to every part of the face, head, neck and throat. From this nerve the teeth are supplied. The pneumogastric nerve, presiding over the functions of the stomach, lungs and heart, receives a branch from this source. Thus it will be seen that a web or net-work of nerves is spread over the entire head, face, neck, throat, lungs, heart and stomach, penetrating their entire tissues, regulating their functions and uniting all in one intimate bond of sympathising action. Now who can be surprised to find that tooth-ache is one of the most distressing, unbearable pains that flesh is heir to?

The most common cause of tooth-ache is from decay in the crown of the tooth, which extends till it has penetrated the canal in the centre of the tooth, and exposes the central pulp above described.

This pulp is one of the most exquisitely sensitive textures in the whole body, and quickly becomes inflamed when exposed to the irritation of foreign substances.

Being confined within the firm sides of the tooth it cannot expand like other inflamed tissues, hence the exquisite pain; for the pain in an inflamed part is very much in proportion to the pressure made upon its nerves by the excessive fullness of the blood vessels. In this state, the individual not only loses the use of the diseased tooth, but most of the rest on that side of the mouth, for he cannot masticate upon any of the teeth near it without danger of pressing the food into the cavity, irritating the nerve and producing tooth-ache.

The nerve of a tooth thus exposed will die of itself if let alone. Some caustic substance that will always destroy living flesh when kept in contact with it, inserted into the cavity will destroy the nerve.

But it always destroys the whole nerve. Every living fibre and filament distributed throughout the minute tubes above described, perishes with it, excepting the short fibres on the outside of the fang, supplied by the periosteum, and these only penetrate a very little way. Within forty-eight hours after the application of the caustic, the tooth will begin to assume a dark hue, from which it never recovers. We have now a dead tooth, discolored and unsightly, in the place of the living semi-transparent pearly gem it was while the nerve was alive. The absorbents that carry off dead and useless matter in contact with living parts, cannot reach the gangrenous fibres, distributed as they are in these minute tubes through the entire substance of the tooth. Hence the continued discoloration of the tooth.

The death of the tooth is immediately followed by inflammation in the socket. The thin membrane which connects the tooth with the socket thickens, and the tooth raises from its place, so that the opposite jaw strikes it in closing the mouth. It is exceedingly tender to the touch. Cold or hot drinks cause severe pain. The adjoining teeth become sore and loosen in the socket. In about a week the inflammation subsides, the tooth gradually settles to its place. A delusive quiet now ensues. The cavity in the tooth may now be filled without pain, other than what is caused by soreness in the socket when pressed upon. The nerve killer can now assure his patient that the cure is complete, and retire from the scene.

Death is repulsive in all its forms. Living textures shrink instinctively from contact with it. The tooth in this state is dead and bears the same relation to living tissues as a splinter of wood, and is only held in its place by the periosteun. A series of efforts now follow from the surrounding parts to expel it from the system. Inflammation first caused in the destroying nerve does not wholly subside. A sack is soon formed around the end of the root, but before this can take place, the jaw bone around the point of the fang has to be removed by absorption to make room for it. Into this sack matter constantly exudes from its inner surface. The absorbents in their turn are able to remove it, while the system is healthful, but so soon as the individual takes cold, a violent inflammation arises in the socket, matter forms so much faster than the absorbents can remove it, that the pressure becomes intense, causing first a dull pain, increasing slowly, aching and throbbing till all the face on that side becomes more or less affected. The sufferer can now trace the anatomical connexion between the nerves of the teeth and the rest of the face. If situated in front, the lip, root of the nose, and the eye are swollen and intensely painful. If at the side, it affects the temple and cheek. If back at the angle of the mouth, the ear, neck and throat swell and are painful, the muscles of the jaw become stiff, and almost close the mouth; head-ache, with fever and throbbing along the course of the sympathising nerves continue without intermission.

The removal of the tooth at this stage will arrest this wild commotion, and shortly restore the patient to health. When left to its course, day after day of the most severe suffering continues, till the matter, which first formed at the point of the fang, can make its way through the side of the jaw bone, or else lift the tooth up in the socket, until it forces a passage out by the side of the fang and bursts upon the gum.

All this is but an effort of nature, to expel the dead tooth from its connexion and contact with the living textures. The gum boil subsides, and the tooth settles down again to its place, but not in peace. A continual warfare is kept up. The Roman Senate were not more intent upon the expulsion of Hannibal from Italy, than are the living textures to be rid of a dead tooth. The sack remains, a fistulous opening upon the gum gives exit constantly to offensive purulent matter. This vile product mingles with the saliva, and with the food, is swallowed with it, and is absorbed into the blood, to taint it, contaminate the secretions and disturb the healthful functions.

Few persons have even a remote conception of the amount of matter that oozes out of these fistulous openings, and mixes with the food in a single day. Then its exceedingly offensive character. Every practitioner of medicine knows that the odor of matter issuing from a diseased bone is among the most disagreeable that he is called to encounter in the whole range of surgery. Then its effect upon the breath. In conversation this taint is imparted more or less to every word that flows from the lips. This matter is not neutralized by its mixture with blood, it is still foreign to the system, and every tissue it comes in contact with, in the course of circulation, repels it till it is thrown out of the system by secretion and exhalation.

The amount of fluid thrown out of the system by exhalation from the lungs, and the skin is much greater than is generally supposed. A mouth that contains three or four of these dead teeth, has as many fistulous abscesses upon the gums pouring out this offensive matter, nearly all of which mixes with the saliva and food, and is swallowed, has a constant source from which the blood is contaminated.

The moisture in the breath is an exhalative from the blood circulating in the lungs, so that the *breath is tainted* with this offensive odor whether it passes through the mouth or not.

Next to the lungs the whole surface of the body is constantly throwing out, either by the sensible or insensible perspiration, the foreign matter contained in the blood.

Persons of neat and cleanly habits would be shocked at the idea of carrying a contaminated atmosphere with them, yet this is true, to some extent, of every person who carries these dead teeth in the mouth.

The evil does not stop here. This contaminating matter passes out through the pores of the skin, and is liable to soil the complexion. This is a more serious evil than many suppose. The nerves of the skin are endowed with a high degree of sensibility; any acrid matter constantly passing out through these minute vessels cannot fail to irritate them, and produce discoloration of the skin, and in many instances to cause eruptive diseases. The little pimples upon the face are often only the inflamed pores of the skin.

It is generally understood that a fine complexion indicates good health, it also implies that the teeth are healthful. I will here say to the fair portion or my audience, that if you keep dead teeth in your mouth, you will do it at the expense of your complexion, your health and your beauty. I have dwelt longer on this topic because these effects upon the constitution seem to be passed over by writers upon the teeth. The inducements to keep dead teeth in the mouth, arising from the na'ural dread that every body feels at the idea of extraction, was great enough before the nerve killing practice was introduced. I feel that it is the duty of the profession to set the evils of this practice fully before the public mind, that all the results of killing the nerve of a tooth and retaining it in the mouth may be known.

